# Magnetic nanotherapeutics for dysregulated synaptic plasticity during neuroAIDS and drug abuse

**DOI:** 10.1186/s13041-016-0236-0

**Published:** 2016-05-23

**Authors:** Vidya Sagar, Venkata Subba Rao Atluri, Sudheesh Pilakka-Kanthikeel, Madhavan Nair

**Affiliations:** Department of Immunology, Center for Personalized Nanomedicine/Institute of NeuroImmune Pharmacology, Herbert Wertheim College of Medicine, Florida International University, 11200 SW 8th Street, Miami, FL 33199 USA

**Keywords:** HIV/AIDS, Morphine, Nicotine, Methamphetamine, Bath salt, Cocaine, HDAC2, BDNF, Neuropathogenesis, Blood-brain barrier, Synaptic plasticity, Magnetic nanoparticles

## Abstract

The human immunodeficiency virus (HIV) is a neurotropic virus. It induces neurotoxicity and subsequent brain pathologies in different brain cells. Addiction to recreational drugs remarkably affects the initiation of HIV infections and expedites the progression of acquired immunodeficiency syndrome (AIDS) associated neuropathogenesis. Symptoms of HIV-associated neurocognitive disorders (HAND) are noticed in many AIDS patients. At least 50 % of HIV diagnosed cases show one or other kind of neuropathological signs or symptoms during different stages of disease progression. In the same line, mild to severe neurological alterations are seen in at least 80 % autopsies of AIDS patients. Neurological illnesses weaken the connections between neurons causing significant altercations in synaptic plasticity. Synaptic plasticity alterations during HIV infection and recreational drug abuse are mediated by complex cellular phenomena involving changes in gene expression and subsequent loss of dendritic and spine morphology and physiology. New treatment strategies with ability to deliver drugs across blood-brain barrier (BBB) are being intensively investigated. In this context, magnetic nanoparticles (MNPs) based nanoformulations have shown significant potential for target specificity, drug delivery, drug release, and bioavailability of desired amount of drugs in non-invasive brain targeting. MNPs-based potential therapies to promote neuronal plasticity during HIV infection and recreational drug abuse are being developed.

## Background

### HIV mediated neurotoxicity

**Fig. 1:**
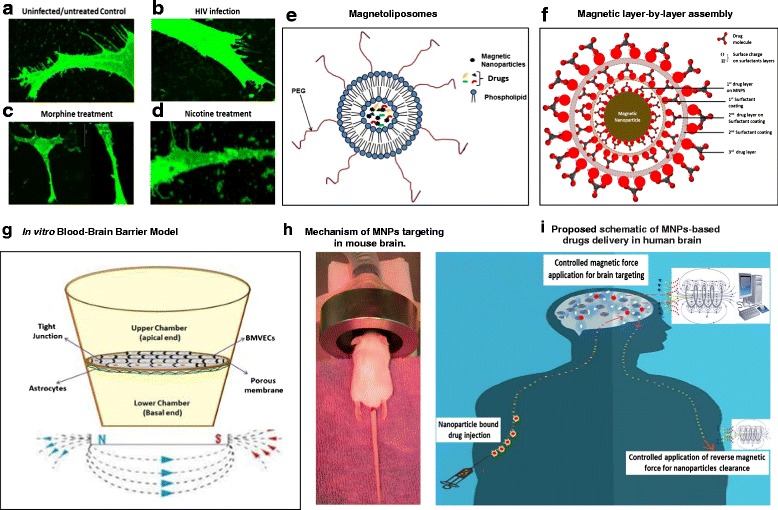
**a**–**d**- Confocal microscopy image showing changes in dendritic and spinal morphology of uninfected/untreated (**a**), HIV infected (**b**) [64], Morphine (**c**) [76], and Nicotine treated (**d**) [62] neuroblastoma (SKNMC) cells; **e**-**f**- Types of magnetic nanoformulations: Magnetoliposomes (**e**) [11] and Magnetic layer-by-layer assembly (**f**) [72]; **g**- In vitro BBB model for : Astrocytes-Endothelial cells co-culture in vitro BBB model: Culture plate is bi-compartmentalized via a transwell porous membrane. The top and underside of this membrane is cultured respectively with tightly junctioned endothelial cells and astrocytes which correspondingly mimics the external (peripheral blood side) and internal (brain microenvironment side) surface of BBB. Magnetic force is applied at the bottom of transwell which influence the transmigration of magnetic nanoformulations [11] (**h**)- Mechanism of MNPs targeting in rodent model: Anesthetized mouse can be placed in a platform with their head positioned between the poles of magnetic coil and retained in the desired field for desired time period. **i**- Proposed schematic of MNPs-based drugs delivery in human brain: Under the influence of in silico-controlled, non-invasive magnetic force from exterior, drug loaded magnetic nanocarriers can be directly transported across the BBB. Drug release at target is mediated by manually uncontrollable, cellular responses such as change in temperature, pH, intracellular Ca_2_^+^ level, etc. or by externally controlled mechanism such as magneto-electric force, radio-frequency magnetic force, etc. Leftover MNPs biodegrades automatically in 2–3 weeks without negative physiological implications in brain or may be cleared immediately by applying reverse magnetic force

It was believed that HIV can enter into the brain only in the final phase of infection when viral load is higher. However, many studies show higher HIV concentration even during the initial infection or shortly after seroconversion [1, 2]. In fact, presence of HIV-proteins, HIV-DNA, and HIV-particles in the brain along with the CNS intrathecal production of anti-HIV antibodies are seen during the initial infection [2, 3]. This substantiates the belief that HIV may sneak into the brain from the beginning of infection. Mononuclear phagocytes, i.e. monocytes and blood-borne macrophages, are the major carriers of HIV into the brain [4]. HIV-infected monocytes from blood stream migrate into the brain in response to specific cytokines/chemokines (e.g. monocyte chemotactic protein-1) [5]. Initial infection of HIV in the brain triggers production of factors that alter the integrity of the blood brain barrier (BBB) (e.g. matrix metalloproteinase) and influence leukocytes transmigration across this barrier [6]. These intensify the HIV infection in various brain cells. Also, differentiation of HIV-infected monocytes into macrophages elicits neuroinflammation by activating astrocytes and resting microglia [7]. Infection and/or immune activation of macrophages and microglia release neuron-damaging products such as TNF-α, IL-1β, reactive oxygen species, nitric oxide, and quinolinic acid, [8, 9]. Additionally four viral proteins, gp120, Tat, Nef, and Vpr have been shown to induce significant neurotoxicity and associated pathology [10]. These HIV proteins can be toxic across various brains cells including neurons (Fig. 1a-b) [11]. The HIV envelope protein gp120/gp41 incites activation of chemokine receptors (CXCR4 or CCR5) on neurons and triggers elevation of intracellular Ca^2+^ leading to apoptosis [12]. Similarly, gp120 activates NMDA receptors in neurons and downregulates glutamate uptake by astrocytes causing excitotoxicity [13]. HIV gp120 also induces nitric oxide synthase production by astrocytes causing cell death [14]. In macrophages and microglia, gp120 induces production of proinflammatory factors such as TNF-α, IL-1β, arachidonic acid, β-chemokines, etc. [15, 16]. Interestingly, gp120 also induces apoptosis in brain microvascular endothelial cells (BMVECs) [17] and inhibits proliferation and migration of neural progenitor cells (NPCs) [18]. Activation of apoptotic p53 pathway by gp120/gp41 has been reported in neurons, astrocytes, and macrophages/microglia [19, 20]. The HIV Tat protein induces multiple effects on neurons: it promotes insertion of NMDA receptors [21], activates NO and calcium release [22], inhibits tyrosine hydroxylase [23], and decreases dopamine [24] which eventually leads to cell death by apoptosis or other cytotoxicity means. In astrocytes, Tat causes upregulation of MCP-1 [24] and diminishes glutamate uptake [25]. Similar to gp120/gp41, Tat in macrophages and microglia induces production of proinflammatory factors such as TNF-α and IP-10 [26]. HIV Tat exposure in BMVECs causes apoptosis induction [27] and in NPCs, neurogenesis is inhibited due to Tat [28]. The HIV Vpr protein induces apoptosis in different brain cells such as neurons [29, 30], astrocytes, and BMVECs [31]. In neurons, Vpr also modulates ion channels [32] and H_2_O_2_ upregulation [33]. Exposure of Vpr to NPCs causes impaired maturation of neurons and mitochondrial dysfunction [34]. The HIV Nef also induces apoptosis in neurons, astrocytes [35], and BMVECs [36]. Additionally, Nef modulates [K^+^] channels in neurons [37] and induces production of proinflammatory factors such as TNF-α, IL-6, MIP-1, and superoxide release in macrophages and microglia [38]. In astrocytes, Nef has been also shown to upregulate complement factor C3, MCP-1, IP-10, and MMP-9 activity. Thus, injury of brain cells by HIV and its proteins involve various cell-specific mechanisms [39].

### Role of drugs of abuse in HIV induced neurotoxicity

Similar to HIV neurotoxicity, recreational drugs alter brain hemostasis and subsequently damage the CNS. Neurotoxicity produced by recreational drugs is analogous to HIV infections and many of them have been shown to promote HIV infections and associated neuropathogenesis (Fig. 1c-d). In fact, it has been shown that HIV proteins and drugs of abuse can exert additive neurotoxic effects. All kinds of recreational drugs including cannabinoids, methamphetamine, cocaine, opioids, etc. have been shown to positively regulate HIV-associated neuropathogenesis [40–45]. Cocaine promotes HIV infectivity in multiple ways with its prime effect on CCR5 upregulation [46]. In synergy with HIV Tat and gp120, cocaine exacerbates neuronal apoptosis via ROS production and subsequent activation of the caspase-3 and NF*k*B pathway [47, 48]. Also, cocaine disrupts BBB permeability which causes influx of HIV infected mononuclear phagocytes into brain [49]. Methamphetamine is another major comorbid factor in HIV-induced neuropathogenesis. It has been shown that methamphetamine and HIV exert an adverse-additive effect on neuronal and glial markers and exacerbate the neurocognitive impairments in methamphetamine-abusing HIV patients. Methamphetamine disrupts dopamine levels resulting in oxidative damage of neurons and has damaging effects on mitochondria of astrocytes [50]. The immunomodulatory effects of opioids have been shown on several hematopoietic cell populations. Opioids induce the expression of μ and other chemokine receptors in monocytic cells resulting in increased HIV susceptibility and stimulation of HIV expression [51]. Also, opiates enhance the production of proinflammatory factors like MCP-1, RANTES, IL-6 and ROS in brain cells [52]. These exacerbate the preexisting inflammation of neurons due to HIV infections. Additionally, changes in the level of endogenous opioids causes disruption of dopaminergic functions which affect the neuro-immunological ability of nervous system to respond against HIV [53]. Cannabinoids uptake significantly suppresses the functional activities of immune cells via activation of cannabinoids receptors, primarily CB2. Various studies suggest a link between cannabinoid-mediated immune suppression and greater susceptibility of HIV infection. As such, it has been proposed that CB2-specific agonists may be useful agents against neuroinflammation [54]. Similarly, alcohol exposure alters the BBB, which lead to increased HIV entry and ROS level in the brain via influx of macrophages [55]. Thus, recreational drugs combined with the virus infection results a unique level of immune incompetence.

## Synaptic plasticity dysregulation and magnetic nanoparticle based therapeutic approach during neuroAIDS

### Synaptic plasticity. Changes during neuroAIDS and drug abuse

Neuronal synaptic plasticity in this context refers to any injury-stimulated changes in neuronal processes such as spine formation, dendritic spines, and synaptic network reorganization. It has been suggested that HAND symptoms in HIV patients are primarily caused by synaptodendritic injury [56, 57]. As such, subjects with HAND exhibit decreased synaptic and dendritic density, resulting from apoptosis and atrophy of grey and white matters [58]. Dendritic spines are postsynaptic specializations which play a critical role in neuronal plasticity. A recent study by Atluri et al. [59] showed significant loss of spines, spine density, dendrite diameter, total dendrite, and spine area in neuroblastoma cells infected with HIV-1 clade B and clade C infected. Inter-clade variations in the density and morphology of spines and dendrites were also noted [59]. Cells infected with HIV1-clade B resulted in a 2–2.5 fold decrease in dendrite diameter, total dendritic area, and spine density. Similarly, a nearly fivefold decrease in the spine length and spine area was found in clade B infected cells compared to uninfected cells. The HIV clade C was found to be less injurious to spine and dendritic phenotypes than Clade B; nonetheless injury was marked when Clade C was compared to uninfected cell culture. This inter-clade variation can be attributed to differences in the potency of neurotoxic peptides of clade B and clade C. As an example, Samikkannu et al. [60] reported that Tat peptide of HIV-1 clade B and C exert different effects on morphology and spine density, with clade C Tat being less potent.

One of the major phenotypic consequences of synaptic plasticity dysregulation is loss of memory. Immediate events recalled via short term memories are consolidated into long term memory for later recall by the brain. Memory consolidation is equally dependent on the changes in physical appearance of neuronal synapses and associated gene expression changes. Immediate-early genes (IEGs), long-term potentiation (LTP) genes, and long-term depression (LTD) genes are three major groups of genes playing central role in synaptic plasticity regulations [59]. It is believed that IEGs mediate LTP to enhance synaptic strength and consolidate memories. Gene expression changes associated with LTD regulate changes in the neuronal synapse that recycle receptors and either enhance or inhibit synaptic connections [61]. Changes in these genes either enhance or depress the synaptic strength causing synapse remodeling. Atluri et al. [62] reported that HIV clade B infection of neuroblastoma cells result in downregulation of 28 major synaptic plasticity genes (ADCY1, ADCY8, BDNF, CAMK2A, CDH2, CNR1, CREM, EGR4, GABRA5, GRIA1, GRIN2A, GRIN2B, GRM1, GRM3, GRM4, GRM7, NCAM1, NFKB1, NOS1, NTF3, NTRK2, PPP1R14A, PRKCG, PRKG1, RELN, RHEB, TIMP1, and TNF), while 8 genes were upregulated (RAB3A, PPP2CA, PIM1, NFKBIB, IGF1, GRM5, GRIN1, and GRIA4). Treatment of neuroblastoma cells by HIV Tat resulted in similar results; nonetheless upregulated or downregulated gene sets in this case were different in comparison to HIV infection [60]. This may be due to differences in neurotoxic potency of HIV particles and individual HIV neurotoxic peptides. Similarly, HIV infection to astrocytes resulted in upregulation of 5 genes (EGR2, EGR4, HOMER1, INHBA, and SYNPO) and downregulation of 28 genes (ADAM10, AKT1, ARC, CAMK2A, CDH2, CEBPB, CEBPD, CNR1, CREB1, DLG4, EGR1, FOS, GABRA5, GRIA1, NMDAR1, GRIN28, GRM1, GRM8, JUN1, JUNB, MAPK1, NF_K_BIB, NGFR, NPTX2, PICK1, PLCG1, PPP1CA, PRKCA, RELA, SIRT1, and SRF) [59]. Cell based variation in the expression of synaptic plasticity genes during HIV infection may reflect infectivity intensity where one cell type establishes latent HIV infection while other is suitable for active infection. Furthermore, as discussed above, addiction of recreational drugs is the major complicating factor for neuroAIDS. Approximately, it is estimated that 13.1 % of the total number of people who inject drugs are living with HIV [63]. Various studies suggest that recreational drugs potentiate the effects of HIV infection in reducing the neuronal plasticity. For example, treatment of nicotine during HIV infection in neuronal cells resulted in downregulation of 47 genes following a combined treatment of nicotine and HIV (compared to 23 genes following HIV alone). Also, spine density was reduced during co-treatment of nicotine with HIV infection [62]. Similarly, Sagar et al. [64] showed significant reduction in spine density following exposure to morphine in the presence or absence of HIV infection. Methamphetamine co-treatment of neuronal cells during HIV infection resulted in additive downregulation of at least 19 genes and lower spine density [65]. A negative effect of methylenedioxypyrovalerone, the main component of bath salt, a synthetic recreational drug, has also been reported where several genes including JUN, JUNB, FOS, RAB3A, PPP1CA, etc. are significantly dysregulated following its acute and chronic treatment. Significant reduction in the spine length, numbers, density, and dendrite diameters of SKNMC cells were also seen after methylenedioxypyrovalerone treatment [66]. The effect of other recreational drugs such as cocaine, alcohol, etc. on synaptic plasticity genes at cellular level has been little studied, nonetheless, brain tissue atrophy and a reduction in neurocognitive performance has been reported in some studies [58].

### Magnetic nanoparticle based therapeutic approaches

MNPs have been extensively investigated for target-specific drug delivery. Nonetheless, MNPs applications for brain drug delivery have been little explored. MNPs possess distinct advantage over other counterparts (Table 1) such as liposomes, micelles and polymeric nanoparticles in that its inherent superparamagnetism allows control over magnetization and therefore the movement and speed of MNPs can be regulated by an external magnetic field. Thus, magnetic nanoparticle based therapeutical formulations can be distributed to specific body locations by applying non-invasive magnetic force (Fig. 1g-i). Importantly, techniques such as magnetic resonance imaging can be used for quantifying localized MNPs-associated drugs which may help in determining site-specific optimal or suboptimal dosing. Another important facet of MNPs which makes it suitable for brain targeting is the flexibility in size. Super paramagnetic iron oxide nanoparticles of ≤10 nm can be synthesized which can cross the BBB without affecting its integrity [67]. In the same line, the basement membrane protein mesh at the BBB has been shown to allow diffusion of targeted immunoglobulins via transcellular transport. Moreover, MNPs can be hybridized with liposomes to get “magnetoliposomes” (Fig. 1e). The magnetoliposomes protect encapsulated drugs loaded on MNPs from biological degradation, reduce the clearance and entrapment of nanocarriers by the reticuloendothelial system, and increase drug bioavailability. Magnetoliposomes can also be utilized for the monocytes/macrophage-based nanodrug delivery at inflammatory sites including the brain [68]. Recently, we have shown the successful delivery of magneto electric nano carrier (MENC) across the BBB into the mouse brain model without significant toxicity [69].

**Table 1:** A comparison of various nanoparticle systems: all of these systems are in preclinical stages for targeted delivery of anti-retroviral and/or anti-addiction drugs to the drug-impenetrable physiological barrier and more rigorous research-homework (particularly in vivo) has to be elucidated to sort out various associated shortcomings

Nanocarriers	Current research standings	Technical limitations and potential improvements
Dendrimers	✓ Preclinical: in vitro BBB model shows increased transmigration of therapeutics; however, yet to be supported by in vivo transendothelial migration assay.	✓ Synthesis process is complex and drug release is inconsistent or premature as well.
		✓ Polycationic moieties of dendrimers induce cytotoxicity and as such, its toxicity on various brain cells must be well defined.
Polymers	✓ Preclinical: In vitro and mouse model studies shows increased transendothelial migration of therapeutics.	✓ Induces transient inflammation and found less ideal for delivery of polar/ionic compounds. As such occurrences of adverse effect, if any, on neuronal cells must be defined and potential of natural polymers should also be explored.
Liposomes	✓ Preclinical: In vitro and rat model studies shows increased migration of therapeutics across BBB.	✓ Drug entrapment ability, in general, is low and it worsens for the water-soluble drugs. Further, drug leaching and carrier instability during storage is also a concern.
		✓ Surface modifications such as PEGylation improves the inherent poor stability of conventional liposomes and can also reduce their uptake by reticuloendothelial system resulting in improved bioavailability. Further, it can be developed as “Trojan nanocarrier” residing in the monocytes/macrophages which naturally transmigrate across BBB.
Solid-lipid	✓ Preclinical: in vitro BBB model shows increased trans-endothelia migration of therapeutics.	✓ Although natural ability of lipophilic material (building block of Solid-lipid nanoparticles (SLN)) to cross the BBB makes SLN a favorable carrier for brain drug delivery, in vivo trans-endothelial migration studies are required to authenticate its applicability.
Micelle	✓ Preclinical: In vitro and mouse model studies shows increased migration of therapeutics across BBB.	✓ Intrinsic nature of particles instability cause premature drug release. In this regard, neuronal cells specific ligand tethering on surface of nanocarrier may improve the active brain targeting.
Magnetic	✓ Preclinical: in vitro BBB model shows increased trans-endothelial migration of therapeutics and several in vivo study show successful brain delivery of MNPs.	✓ Limited in vivo study showing site-specific targeting and lab-to-land transfer ability for anti-retroviral and anti-addiction drugs.
	✓ Advantages over other nanoparticles: Movement and speed of nanocarrier can be controlled by external magnetic force which helps in escape of nanocarriers’ uptake from reticuloendothelial system and subsequently accelerated active targeting and increased bioavailability is achieved.	✓ Also, MNPs can be hybridized with liposomes as “Magneto-liposomes” for development of magnetized “Trojan nanocarrier” residing in the monocytes/macrophages. While monocytes/macrophages can naturally transmigrate across BBB, presence of “magneto-liposomes” in its cytoplasmic space can add to its movement influenced by external magnetic force.

A variety of biomolecules such as proteins, enzymes, and synthetic drugs can be immobilized on the exterior of coated or uncoated MNPs and can be guided magnetically to targeted sites [70]. However, the application of MNPs for treatment of drug abuse and neuroAIDS is limited. In recent years, our laboratory has intensively studied the transendothelial delivery of MNPs-bound anti-retroviral (ARV) and anti-addiction drugs. ARV drugs such as 3^′^-azido-3^′^ -deoxythymidine-5^′^ -triphosphate (AZTTP) and anti-morphine drugs such as CTOP could be directly (with no additional coatings) immobilized on the MNPs surface. It was found that the efficiency of AZTTP-magnetic nanoformulations, as determined by suppression of HIV replication, remains comparable to the free drug. The AZTTP-magnetic nanoformulations were also hybridized with liposomes which could sustainably release the drugs for 14 days with intact potency in inhibiting HIV replication [68]. Importantly, under the influence of an external magnetic field, both AZTTP-magnetic and AZTTP-magnetoliposome nanoformulations could have significantly higher (nearly 3 fold) transmigration across in vitro BBB, compared to free AZTTP, without affecting the integrity of the BBB. A similar result was obtained with AZTTP immobilized on magneto-electric nanoparticles (MENPs), when p24 inhibition efficiency of free drugs was compared to a.c. triggered released drugs from MENPs [71]. The sustained release nanoformulations developed by Jayant et al. [72] was effective in loading 2.8 times more Tenofovir and it increased the drug release period by 30-fold. This layer-by-layer nanoformulation of dextran sulphate bilayer on MNP, sandwiching anti-HIV drugs in between, showed better blood–brain barrier transmigration ability (37.95 % ± 1.5 %) and in vitro antiviral efficacy (~33 % reduction of p24 level) over a period of 5 days after HIV infection in primary human astrocytes. Fiandra et al. [73] showed that PMA coated ultra-small MNPs (10 nm) improve the permeation of Enfuvirtide across both in-vitro BBB and mouse models. Wen et al. [74] explored the transendothelial migration potential of a magnetoliposome nanoformulation with or without Tat coating. A dose and time dependent increase in the accumulation of Tat-coated magnetoliposome was seen in the endothelial cells. It was found that Tat-conjugated magnetoliposome may be an effective brain drug delivery system [74]. Raymond et al. [75] explored MNP based targeting to block the release of Nef containing exosomes from microglia in in vitro system. This approach showed a positive impact in protecting in vitro BBB integrity and permeability from Nef-exosome toxicity and we speculate that this technique may be beneficial in preventing or reducing HIV-associated neuropathogenesis [75]. All of these anti-retroviral nanoformulations show great promise in reducing or eliminating HIV load from the brain. As such magnetic nanoformulations of other ARV drugs can be developed. Nonetheless, ARV alone can only prevent the expansion of HIV mediated damage and may not be sufficient to revive or promote already damaged neuronal plasticity during HIV infection. Additionally, synaptic plasticity dysregulation by illicit drugs is accelerated during HIV infection and vice-versa (see above). Thus, an ideal therapeutic approach for treatment of synaptic plasticity dysregulation during neuroAIDS should also be able to revive and promote neuronal plasticity even when illicit drugs are present [58]. In this context, a magnetic nanoformulation of morphine antagonist (CTOP) has been developed by our group. Confocal microscopy study revealed that the efficacy of CTOP-magnetic nanoformulations was comparable to free CTOP in protecting modulation of neuronal dendrite and spine morphology during morphine exposure and morphine-treated HIV infection [64]. Similarly, Pilakka-Kanthikeel et al. [76] investigated a magnetic nanoformulation of a major neuron-resuscitating agent, BDNF. The BDNF-magnetic nanoformulation was able to revive the morphine induced degradation of spine density. Since HIV infection also reduces BDNF expression, BDNF-magnetic nanoformulations could be a common therapeutical agent for both neuroAIDS and drug addiction mediated neuronal plasticity dysregulation. In fact experimental evidence support BDNF as a therapy for HAND. Similarly magnetic nanoformulations of other agents, which could be beneficial for synaptic plasticity during neuroAIDS and drug addiction, could be developed. For example, studies have shown a neuroprotective role of platelet-derived growth factor via the induction of the synaptic plasticity gene Arc. Methanandamide, a synthetic stable analog of the endocannabinoid anandamide, has been demonstrated to improve motor function and downregulates the production of inflammatory mediators in microglial cells [77]. Similarly, a role for HDAC2 and miR-485 in regulation of synaptic plasticity genes in HIV infection has been shown [78]. Additionally, HIV latency breaking agents such as vorinostat and bryostatin could be beneficial [62]. Thus, magnetic nanoformulations of these agents, either singly or in combination, could be used to promote synaptic plasticity during neuroAIDS and drug addiction. The cell viability of the magnetic nanoformulations mentioned above has been >95 %, making them potentially promising therapeutic agents. However, much research is required before the practical application of this approach in a clinical setting.

### MNPs bioavailability and drug release at target

**Fig. 2:**
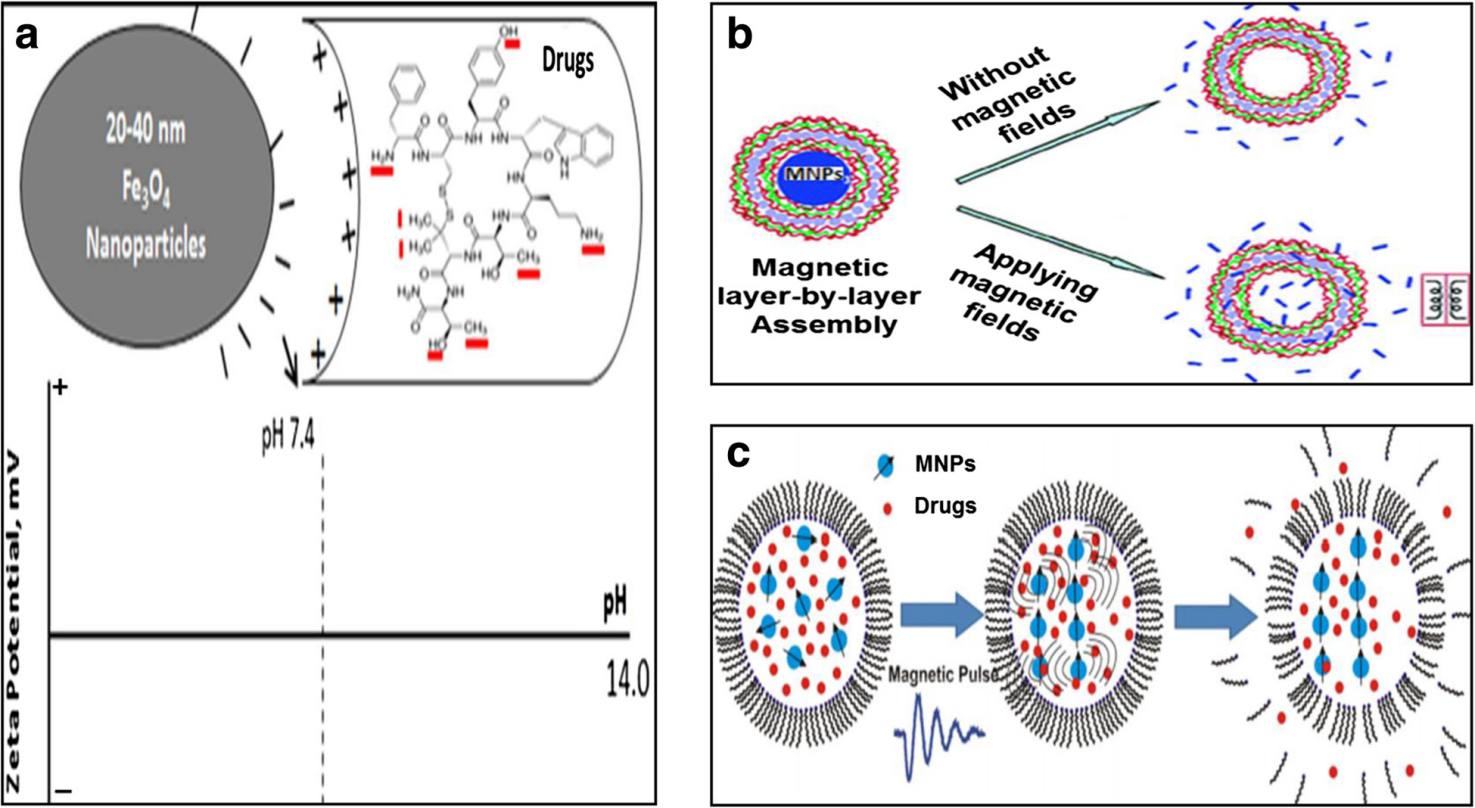
**a** - Schematic illustration showing process of drug loading on MNPs. Interaction between negative charge on MNPs surface and positive charge on different moieties of drug molecules influence drug binding on MNPs [62]. **b**-**c**-Geometry of magnetic-polymer layer-by-layer (**b**) and magnetoliposomes (**c**) assembly under magnetic field: Application of external magnetic field generate torque and magnetic pulse in the encapsulated MNPs such that external layer of carrier is disturbed and distorted. This results in increased carrier permeability [84, 85]

A major advantage of MNPs mediated drug delivery lies in the quick delivery and early availability of associated drugs at the targeted site(s) in comparison with contemporary nanocarriers. This is achieved by an externally applied magnetic force which accelerates MNPs-drug delivery to the target site. The movement of all MNPs based nanocarriers such as magnetoliposomes or magnetized monocytes/macrophages can be manipulated in the same way as for naked MNPs. For drug delivery into brain and exposure of magnetic fields, anaesthetized animals are generally injected intravenously, and subsequently placed on a platform with their head positioned between the poles of magnetic coil and retained in the field for the desired time (Fig. 1h-i). Several rodent models have been developed in recent years to study neuroAIDS transmission and pathogenesis [79]. Kong et al. [80] have shown that accumulation of 124 nm MNPs in mouse brain reaches its peak between 15 min and 2 h with an external magnetic field intensity of ~1000 Oe. Notably, MNPs level in brain slowly diminished over time and vanished in 2–3 weeks without negative physiological effects [80]. Unlike controlled and targeted delivery of MNPs, release of drugs from MNPs cannot be controlled manually. Fe_3_O_4_/Fe_2_O_3_ or its derivatives are the most commonly used MNPs in biomedicine. These MNPs have an inverse spinel cubic crystal lattice where face-centered cubic lattice are occupied by O^2−^ anions and tetrahedral and octahedral sites are occupied by Fe^2+^ or Fe^3+^ anions. Octahedral Fe moieties are primarily involved at environmental interfaces and in an aqueous medium the OH group of water molecules cause protonation (Fe-OH + H^+^ = Fe-OH_2_^+^) and deportation (Fe-OH = Fe-O^**−**^ + H^+^), resulting in positive or negative charged surface depending on the pH of the solution [81, 82]. Drug molecules containing opposite charge moieties can be immobilized on the MNPs surface via ionic interaction in a pH- dependent manner (Fig. 2a). Similarly, OH_2_^+^, O^**−**^ or other moieties on MNPs/drug molecules are generated due to H_2_O reactivity. This tunability in the surface charge allows binding of a wide range of biomolecules to MNPs either via ionic interactions or via surface coating or tethering agents [83, 84]. It is believed that manually uncontrollable, cellular factors such as pathology-specific changes in temperature, pH and intracellular Ca_2_^+^ level mediate drug release by modulating the original ionic interactions of MNPs and drug molecules. Recently we have discovered a novel magneto–electric nanoparticle for targeted drug delivery and on-demand drug release where under the influence of an external AC trigger, the symmetry of ionic bonding (charge distribution) between drug molecules and nanoparticles can be broken, and thus drug release from particles can be controlled as and when required [11, 85–87]. Similarly, silica-coated magnetic nano-capsules allow on-demand drug release via a remote radio-frequency magnetic field [80]. MNPs can also be coated with a near-infrared- photosensitive hydrogel layer which will allow automated navigation and targeted delivery via an external, non-invasive magnetic field and near infrared photo-targeting for sensing and controlled release of drugs [88]. Furthermore, Jayant et al. reported a layer-by-layer (LbL) assembly (Fig. 1f) of dextran sulphate on MNPs for development of sustained release formulation of anti-retroviral drugs [72]. Nonetheless, development of externally controlled drug release mechanisms from MNPs remains a major concern. It is believed that the application of an external magnetic field generates torque in the encapsulated MNPs of magnetoliposomes, magnetic nano-capsules and layer-by-layer assemblies. This effect significantly disturbs and distorts the external geometry of nanocarrier causing increased carrier permeability (Fig. 2b-c).

## Conclusions

Although, highly Active Antiretroviral Therapy (HAART) has resulted in a remarkable decline in morbidity and mortality in AIDS patients, viruses still remain in the brain sanctuary causing neurotoxicity and subsequent HIV associated neurocognitive disorders (HAND). Also, HIV infections potentiate the effects of drugs of abuse in compromising neuronal plasticity (and vice-versa). Delivery of therapeutic agents including anti-HIV and anti-addiction drugs to the brain remains a challenge due to impenetrability of the tightly-junctioned BBB. In this context, nanotechnology promises exciting prospects for the development of a novel drug delivery system to transport the desired therapeutic levels of drugs across the BBB. Among existing nano-based drug delivery methods for brain targeting, only MNPs combine the advantages of target specificity, drug delivery, drug release and bioavailability of the desired drug concentration. Several modifications in the MNPs-based drug carrier have been investigated. While MNPs alone can be sufficient to deliver drugs at target site, magneto-electric particles can allow control over drug release as and when required. Similarly, layer-by-layer sustained release nanoformulations on MNPs can be beneficial in reducing the therapeutics program from a daily-basis to longer intervals. Successful implementation of these therapeutic approaches in clinical settings may provide a step forward towards achieving near-normal life expectancy for HIV and drug addiction patients.

### Ethics approval and consent to participate

As this study did not involve any animal or human participants, human data or human tissue, ethical committee approval is not required.

## Abbreviations

AIDS: acquired immunodeficiency syndrome
ARV: anti-retroviral
AZTTP: 3′-Azido-2′,3′-dideoxythymidine-5′-Triphosphate
BBB: blood brain barrier
BDNF: brain derived neurotropic factors
BMVECs: brain microvascular endothelial cells
CNS: central nervous system
CTOP: D-Pen-Cys-Tyr-DTrp-Orn-Thr-Pen-Thr-NH2
DNA: deoxyribonucleic acid
HAART: highly active antiretroviral therapy
HAND: HIV associated neurocognitive disorders
HDAC: histone deacetylase
HIV: human immunodeficiency virus
IEGs: immediate-early genes
LBL: layer-by-layer
LTP: long-term potentiation
LTD: long-term depression
mir: micro RNA
MNPs: magnetic nanoparticle
NPCs: neural progenitor cells
NO: nitric oxide
Oe: oersted
PMA: poly (methyl acrylate)
ROS: reactive oxygen species
